# The role and mechanisms of cordycepin in inhibiting cancer cells

**DOI:** 10.1590/1414-431X2024e13889

**Published:** 2024-08-23

**Authors:** Gong Yu, Jiahua Peng, Lu Li, Wenbin Yu, Bin He, Bin Xie

**Affiliations:** 1 School of Chinese Medicine, Jiangxi University of Chinese Medicine, Nanchang, Jiangxi, China; 2 Jiangxi Key Laboratory of Bioprocess Engineering, College of Life Sciences, Jiangxi Science & Technology Normal University, Nanchang, Jiangxi, China

**Keywords:** Cordycepin, Cancer, Cells apoptosis, Cell proliferation

## Abstract

With the escalating incidence and mortality rates of cancer, there is an ever-growing emphasis on the research of anticancer drugs. Cordycepin, the primary nucleoside antibiotic isolated from *Cordyceps militaris*, has emerged as a remarkable agent for cancer prevention and treatment. Functioning as a natural targeted antitumor drug, cordycepin assumes an increasingly pivotal role in cancer therapy. This review elucidates the mechanisms of cordycepin in inhibiting tumor cell proliferation, inducing apoptosis, as well as its capabilities in suppressing angiogenesis and metastasis. Moreover, the immunomodulatory effects of cordycepin in cancer treatment are explored. Additionally, the current status, challenges, and future prospects of cordycepin application in clinical trials are briefly discussed. The objective is to provide a valuable reference for the utilization of cordycepin in cancer treatment.

## Introduction

Cancer is a complex disease that involves various factors and mechanisms. Anticancer drugs now target multiple pathways beyond just cell cycle progression and apoptosis induction (1,2). Modern anticancer drugs also focus on targeting specific molecular alterations in cancer cells and immune system evasion (3). This approach allows for personalized and targeted therapies tailored to individual patients (4). Fungal secondary metabolites have been widely applied in cancer treatment for their numerous bioactive compounds (5). For example, neutral glycosylceramides, due to their ability to effectively inhibit the proliferation of cancer cells, have been used as a “functional ingredient” added to health foods (6). Over the years, extensive research about anticancer agents has been conducted on these bioactive compounds, including adenosine, cordycepin (COR) (7), coumarin, and exopolysaccharide. Cytotoxic nucleoside analogues have a long history as chemotherapeutic agents in cancer treatment (8). While nucleoside analogues were indeed used for cancer treatment among the earliest chemotherapeutic agents, significant advancements have been made since then (9). The discovery and development of new classes of drugs, including immunotherapies, have revolutionized cancer treatment strategies.

COR, also known as 3'-deoxyadenosine (10), is a nucleoside antibiotic isolated from *Cordyceps* fungi (11). Much research has demonstrated that COR possesses various pharmacological properties, including anti-tumor, anti-metastatic, anti-inflammatory, anti-oxidative, anti-influenza, and immunomodulatory effects (12). Its profound efficacy manifests in controlling cancer growth and metastasis, making it a suitable adjuvant in post-surgical or post-radiotherapy/chemotherapy interventions for cancer patients. Through *in vitro* studies, COR showed potential in antitumor activity (13). Further studies are needed to confirm and elucidate the potential antitumor activity of COR and its impact on the molecular regulation of fungal secondary metabolism. This research will help uncover the mechanism of action of COR and the molecular regulatory network of fungal secondary metabolism, supporting the development of new therapeutic strategies for cancer treatment.

Previous research has demonstrated that COR has the ability to induce cancer cell apoptosis (14), cause cell cycle arrest, inhibit cancer cell metastasis, and modulate the expression of tumor suppressor genes (15). Furthermore, when combined with anticancer drugs like cisplatin (DDP), paclitaxel, or radiation therapy, COR has been found to enhance the sensitivity of cancer cells to these treatments, thereby augmenting their anticancer efficacy (16). The study conducted by Liao et al. (17) demonstrated that the combination of COR and apatinib has a synergistic anticancer effect on non-small cell lung cancer cells. The combination of COR and doxorubicin has been reported to have significant inhibitory effects on the activity, proliferation, and migration of liver cancer cells and glioblastoma (18,19).

Due to the increasing incidence of cancer worldwide and the limited efficacy of existing therapies, there is a growing need for new and effective treatments for this disease (20). As such, the study of COR in cancer therapy has become an active area of research. Therefore, this review aims to provide a comprehensive overview of the role and mechanisms of COR in inhibiting cancer cells. Additionally, this review provides a comprehensive overview of COR's modulation of the immune system in the context of cancer therapy. We focused on the mechanisms through which COR influences immune cell function, cytokine production, and immune checkpoint regulation. Furthermore, we discussed the preclinical and clinical evidence supporting the use of COR as an immunomodulatory agent in cancer treatment. A thorough understanding of these aspects will facilitate the development of novel immunotherapeutic strategies harnessing the potential of COR for enhanced cancer therapy outcomes.

## Effects of cordycepin on cancer cells

### *In vitro* studies on the impact of cordycepin on various cancer types

The study conducted by Li et al. (21) showcased COR's capacity to effectively inhibit the growth of colon cancer cells *in vitro*. In an *in vitro* study conducted by Khuntawee et al. (22), using the HT-29 colon cancer cell line, COR demonstrated its ability to inhibit cell growth by inducing apoptosis. Treatment with both non-encapsulated and encapsulated COR at a dosage of 125 μg/mL resulted in a significant decrease in cell viability below 50% after 48 h. These findings not only suggest that COR-encapsulated liposomes hold promise as a potent drug candidate for cancer therapy but also highlight the promising potential of COR as a novel therapeutic agent for the prevention of cancer.

To study the effect of COR on apoptosis, Cui et al. (23) performed cell treatment experiments and measured them using a Muse cell analyzer. They underscored the potential of COR as a valuable therapeutic approach, targeting cancer stemness and reversing chemoresistance in ovarian cancer. Tania et al. (24) discovered that the administration of COR effectively controlled the growth of SiHa and HeLa cervical cancer cells *in vitro* and *in silico*. It not only increased the rate of apoptosis but also disrupted the cell cycle, leading to elongation of the S-phase. In the study on human pancreatic cancer cells, the researchers evaluated the antitumor viability of COR using colony formation assays. Through the annexin V/PI double staining and flow cytometry assay, it was revealed that COR induced apoptosis and caused cell cycle arrest in human pancreatic cancer cells (25). These *in vitro* studies provide valuable insights into the potential anticancer effects of COR on different cancer types. However, further research is needed to validate these findings and explore the underlying mechanisms of COR's action.

### *In vivo* experiments on the anticancer effects of cordycepin

Feng et al. (26) conducted a 30-day experiment with COR on mice. The results of the study showed that the combined treatment of anti-cluster of differentiation (CD) 47 antibody and COR significantly reduced the proliferation of melanoma cells in mice and significantly prolonged the survival period of mice. In a study conducted by Sato et al. (27), black melanoma-bearing mice were orally administered with a dosage of 15 g/L of COR. The results showed that COR exhibited a remarkable tumor suppression rate of up to 36% without causing any significant reduction in mouse body weight or systemic toxic side effects. In order to validate the anti-hepatocellular carcinoma action of COR, Zhou et al. (28) conducted engraftment experiments using phospholipase C (PLC)/PRL/5-xenografted BALB/c athymic nude mice. In an *in vivo* study (13), it was observed that the combination of COR with conventional chemotherapy was effective in treating tumors. Chang et al. (29) suggested that COR has a suppressive effect on fibroblast growth factor 9-induced tumor growth in the mouse allograft model. The study conducted by Zheng et al. (30) investigated the *in vivo* anti-tumor activity of COR using a murine xenograft model system. The researchers found that COR has the ability to inhibit the growth of human tongue cancer cells and induce apoptotic death through the mitochondrial pathway. Taken together, COR exhibits the potential to treat tumors *in vivo* and can complement traditional chemotherapy drugs. These findings provide an effective theoretical basis for further exploration and utilization of COR as an anticancer drug. However, more studies are needed to gain a deeper understanding of the mechanism, dose, and effect of COR on other cancer types in order to maximize its antitumor potential and provide a more reliable basis for clinical application.

### Mechanisms underlying cordycepin's anticancer effects

COR demonstrates potential anti-tumor activity against different types of tumors. It has been studied for the treatment of breast cancer, lung cancer, liver cancer, colon cancer (31), gastric cancer, prostate cancer, cervical cancer, and other types of cancer (32). The mechanisms of action of COR are multifaceted; specifically, COR can target signaling pathways such as PI3K/AKT (33), mitogen-activated protein kinase (MAPK), nuclear factor-κB (NF-κB) (23), and Wnt/*β*-catenin, as well as regulate the expression and activity of apoptosis-related proteins such as the B-cell lymphoma-2 (Bcl-2) protein family, Caspase protein family, and poly ADP-ribose polymerase (PARP) (28).

### Cordycepin's role in regulating tumor cell proliferation and apoptosis

#### COR induces cell apoptosis through the Caspase pathway

COR possesses multiple mechanisms for its anticancer properties, with one prominent mechanism being the induction of cellular apoptosis (34). Apoptosis, a programmed cell death process, is essential for normal cell growth and aging in multicellular organisms, the dysregulated apoptosis can lead to various diseases, including colorectal cancer (CRC). Nucleoside analogues among antitumor drugs induce apoptosis through diverse mechanisms, such as cell cycle and replication arrest, transcription regulation, DNA repair, and the modulation of apoptosis and autophagy pathways (35).

**Figure 1:**
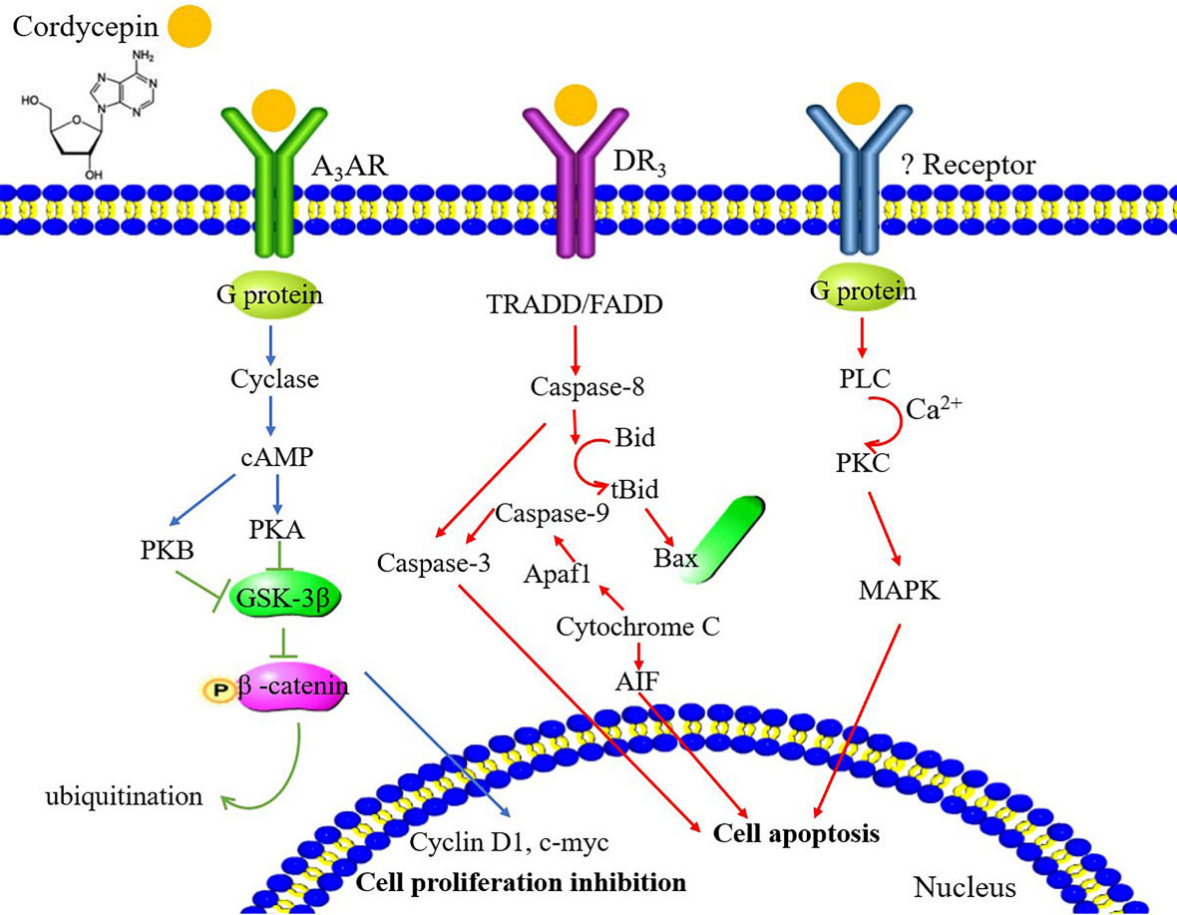
Schematic diagram illustrating the molecular mechanisms by which cordycepin exerts its anticancer effects, including inhibition of proliferation and induction of apoptosis. Blue arrows represent signaling inhibition. Red and green arrows indicate the processes involved in cell apoptosis and proliferation inhibition, respectively. A_3_AR: A_3_ adenosine receptor; AIF: apoptosis-inducing factor; Bcl-2: B-cell lymphoma-2; Caspases: cysteine-aspartic proteases; DR_3_: death receptor 3; FADD: Fas-associated protein with death domain; PKA, B, or C: protein kinase A, B, or C; PLC: phospholipase C; tBid: truncated Bid; TRADD: type 1-associated death domain protein; MAPK: mitogen-activated protein kinase; FADD: fas-associated protein with death domain; G protein: guanine nucleotide-binding protein; Bid: BH3-interacting domain death agonist; Bax: Bcl2-associated x protein; GSK: glycogen synthase kinase.

The effect of COR includes the relocation of the pro-apoptotic protein Bax from the cytoplasm to the mitochondria, the release of cytochrome c into the cytoplasm, ultimately inducing intrinsic apoptosis in cells (14). The research conducted by Tung et al. (36) indicating that through the activation of Jun NH_2_-terminal kinase (JNK) and cysteine-aspartic proteases (Caspases) pathways, COR exhibited a remarkable ability to induce apoptosis in human oral cancer cells. Hwang et al. (37) indicated that COR inhibits the tumor necrosis factor (TNF)-α-mediated NF-κB signaling pathway. This inhibition subsequently triggers the activation of the MKK7-JNK signaling pathway by suppressing c-FLIPL expression, resulting in apoptosis of renal carcinoma cells. Caspases, an essential gene family, play a pivotal role in preserving the dynamic balance within the body by regulating cellular apoptosis and inflammatory reactions (38). Cui et al. (23) conducted a study that demonstrated the ability of COR to inhibit CCL5-mediated Akt/NF-κB signaling, which upregulates Caspase-3 activation in human ovarian cancer cells. Upon the binding of COR to death receptor 3 (DR_3_), the receptor recruits initiator Caspase-8 through the adaptor protein tumor necrosis factor receptor type 1-associated death domain protein/Fas-associated protein with death domain (Figure 1) (39). Subsequently, Caspase-8 undergoes oligomerization and activation via autocatalysis. One of these events involves the activation of Caspase-8, which then cleaves the pro-apoptotic Bcl-2 family protein called Bid (40). Once Bid is cleaved by Caspase-8, it produces a truncated form known as truncated Bid (tBid). This tBid molecule then translocates to the mitochondria, which leads to an increase in permeability of the mitochondrial membrane. This increased permeability causes the release of cytochrome c from the mitochondria into the cytoplasm (41). Cytochrome c has been shown to activate apoptosis-inducing factor (AIF), which translocates to the nucleus and initiates cell apoptosis (34). The release of cytochrome c is a critical step in apoptosis as it activates downstream Caspases, including Caspase-3. Caspase-3 then further propagates the apoptotic signaling cascade, leading to cell death (42). In conclusion, one of the anticancer mechanisms of COR involves the inhibition of cancer cell proliferation through the regulation of Caspase-mediated cell apoptosis.

#### Cordycepin inhibits the cell cycle

To ensure the stability of genetic information and the smooth progression of cell division, multiple checkpoints are present throughout the cell cycle. These checkpoints function to delay the progression of the cell cycle or induce the cell to exit the cycle or undergo programmed cell death in the presence of irreparable DNA damage (43). However, cancer cells have evolved mechanisms to alter their own structure, evade signaling pathways, and bypass cell checkpoints, allowing them to proliferate uncontrollably. Therefore, targeting the cell cycle has been considered a promising therapeutic approach for cancer treatment (44).

Due to its binding to A_3_ adenosine receptor (A_3_AR) and subsequent inactivation of the GSK-3*β/β*-catenin signaling pathway and activation of G protein, COR inhibits the formation of cAMP (Figure 1) (45). cAMP exerts its regulatory role in the cell cycle by modulating key signaling pathways, inhibiting S phase entry, regulating cyclin-dependent kinases (CDKs) activity, and influencing gene expression of cell cycle regulators (46). In response to DNA damage during cell division, the cell cycle will be paused by inhibiting cyclins and CDKs or corresponding protein expression. This allows sufficient time for DNA damage repair and reduces the risk of mutation occurrence (47). COR treatment resulted in the arrest of the human lung cancer cell line at the G0/G1 phase of the cell cycle. Compared to the EGF-stimulated control group, the levels of apoptosis-related proteins Caspase-3 and Bcl-2 decreased. However, there was an increase in the protein expression levels of the proapoptotic protein Bax and cleaved Caspase-3 (48). Additionally, COR inhibits the expression of cell cyclins E and A2, which are crucial for the cell's transition from the G1 phase to the S phase. As a result, COR induces cell cycle arrest by inhibiting the transition from the G1 phase to the S phase in cancer cells (49). Additionally, anti-oral cancer activity of water extract from the mycelia of surface liquid-cultured *Cordycepin militaris* (WECM) was evaluated. The results revealed that WECM caused cell cycle arrest in the G2/M phase (50). The G2/M phase is a critical stage in the cell cycle where cellular components are duplicated and prepared for cell division. The arrest of oral cancer cells in this phase suggests that WECM interferes with the normal progression of the cell cycle, thereby impeding cell growth and proliferation. COR also activates the Chk2 pathway while downregulating cyclin A2 and CDK2 phosphorylation (51). These results suggest that COR shows promising potential as a leading compound for the development of new anti-pancreatic cancer drugs. It inhibits the growth of pancreatic cells by inducing cell apoptosis and causing cell cycle arrest.

CDK1 and cyclin B1 are important regulatory proteins involved in the progression of the cell cycle (52,53). The cell cycle assays demonstrated that COR has the ability to alter the expression of cyclin-dependent kinase 1 (CDK1) and cyclin B1, leading to a blockade of the G2/M phase of the cell cycle in esophageal cancer cells (54). Researchers have discovered that treatment with COR increases the proportion of cells in the G2/M phase while decreasing the proportions of cells in the G1 and S phases in human bladder cancer cells (55). The COR treatment by Joo et al. (56) was found to upregulate caveolin-1 expression, which subsequently activated the JNK pathway and increased the phosphorylation of Forkhead transcription factor. The activated Foxo3a then translocated to the nucleus and triggered the expression of pro-apoptotic genes, ultimately leading to apoptosis in lung cancer cells. These findings indicate that COR can inhibit the proliferation of cancer cells through cell cycle arrest. Similar results have been observed in human breast cancer cells (57). Research has shown that COR pretreatment mitigated the inflammatory response and inhibited apoptosis and autophagy via regulation of the MAPK/NF-κB signaling pathway. COR has been observed to significantly enhance hyperthermia-induced apoptosis and G2/M phase arrest in human leukemia cells. The combined treatment enhanced apoptosis through the MAPK pathway and mitochondrial dysfunction (58). In the study conducted by Pao et al., increased expression of protein kinase C (PKC), extracellular signal-regulated kinase 1/2 (ERK1/2), and c-Jun N-terminal kinase (c-JNK) was observed in mouse Leydig tumor cells treated with COR signaling pathways, leading to cell death in the tumor cells (59). Similarly, Lee et al. observed an upregulation of JNK-inactivating phosphatase in response to COR treatment (60). This upregulation suggests that COR can inactivate JNK, which is a component of the MAPK and downstream PLC/PKC signaling pathways (61). In conclusion, COR can inhibit cell proliferation by regulating the expression of cyclins, CDKs, PLC/PKC, and the MAPK signaling pathway, thus blocking the cell cycle.

#### Cordycepin can regulate the expression of P53

P53 is a protein known as a tumor suppressor gene, which plays an important regulatory role in repairing the changes in the environment and restoring cellular homeostasis, including maintaining normal cell cycle, repairing DNA damage, and inducing cell apoptosis (62). The activation of P53 can induce a series of anti-proliferative responses, including cell apoptosis, senescence, differentiation, and metabolic regulation, which seem to be the main mechanisms by which P53 inhibits tumor formation (63), but the specific mechanisms have not been elucidated.

Although the exact mechanism of P53 as an anticancer target is not yet clear, existing studies suggest that COR inhibits the proliferation of cancer cells by activating the expression of P53. A study suggests that COR increases the expression of P53, promoting the release of cytochrome c from mitochondria to the cytoplasm (49). The released cytochrome c can activate Caspase-9, leading to intrinsic apoptosis in leukemia cells (49). In experiments studying the effects of COR on rat glioma cell lines, researchers found that COR induces cell apoptosis by increasing the expression level of P53 protein in cancer cells, and this induction is blocked by P53 siRNA knockdown, which further confirms the fact that COR induces cancer cell apoptosis by inducing the expression level of P53 (21). Studies have shown that COR can induce apoptosis in various cancer cells, including leukemia cells (49), liver cancer, oral squamous cell carcinoma, endothelial cells, and breast cancer, by regulating the level of P53 expression.

#### ROS as a novel target for cancer treatment

Historically, reactive oxygen species (ROS) have been linked to cancer development. However, recent research has revealed that the effects of ROS on cells can vary greatly depending on their concentration. In fact, current studies indicate that ROS can have both beneficial and detrimental effects on cellular processes associated with cancer, such as proliferation and migration (64). This nuanced understanding of ROS highlights their potential as a target for innovative cancer therapies. Moderate levels of ROS can sustain cancer cell proliferation, migration, and survival (65). Furthermore, the generation of ROS has been shown to significantly enhance the resistance of gastric cancer cells to chemotherapy drugs, while elevated levels of ROS can induce cell apoptosis or necrosis (66). Therefore, extreme levels of ROS can promote cell death, making ROS a novel target for cancer treatment. Kim et al. (67) found that COR induces the overexpression of ROS in human bladder cancer T24 cells, leading to the inactivation of the ROS-dependent phosphoinositide 3-kinase (PI3K)/Akt signaling pathway, thereby inducing T24 cell apoptosis. Dong et al. (57) discovered that the expression level of ROS significantly increases in breast cancer cells after treatment with COR, consequently enhancing DNA damage and leading to cell cycle arrest and apoptosis following radiation stimulation. Research has indicated that ROS expression is elevated in COR-treated cells of brain cancer (68), gastric cancer, and esophageal cancer (54), resulting in the induction of cell apoptosis and the inhibition of cancer cell proliferation.

### Cordycepin's ability to inhibit angiogenesis and metastasis

Angiogenesis refers to the formation of new blood vessels that supply nutrients and oxygen to tumors, while metastasis involves the spread of cancer cells from the primary tumor to distant sites in the body. COR has been shown to possess the ability to inhibit angiogenesis and metastasis, which are key processes in cancer progression. The mechanisms through which COR exerts these effects include modulating the expression of various proteins and signaling pathways (69). Specifically, Dong et al. (57) suggest that COR has the potential to inhibit the migration and invasion of HCT116 cells by modulating EP4 expression and the AMPK-CREB signaling pathway. This indicates that COR could serve as an effective anti-cancer agent in therapeutic strategies targeting colorectal cancer metastasis. According to the experimental results of Nakamura et al. (12), COR exhibits anti-metastatic effects through the following pathways: first, it stimulates the adenosine A_3_ receptor; second, it activates glycogen synthase kinase (GSK)-3*β* and inhibits the expression of cyclin D1 express. In addition, COR also inhibited the platelet aggregation induced by cancer cells, inhibited the activity of matrix metalloproteinase (MMP)-2 and MMP-9, and promoted the secretion of tissue inhibitor of metalloproteinase (TIMP)-1 and TIMP-2, thereby inhibiting invasiveness. The most notable biological features of malignant tumors are invasion and metastasis, which are closely related to the tumor cell microenvironment (70). Research has indicated that the inhibitory effects of COR on retinoblastoma cell proliferation, migration, invasion, and lung metastasis were achieved through the modulation of the c-Myc/cyclin D1 pathway (71). Additionally, treatment of glioblastoma cells with COR resulted in a significant dose-dependent decrease in the expression levels of MMP-2 and MMP-9. Similar results have been observed in gastric cancer (72), and colorectal cancer cells as well (73).

Platelet C-type lectin-like receptor 2 (CLEC-2) can affect the proliferation, migration, and metastasis of tumor cells. After treating gastric cancer cells with COR, the expression of CLEC-2 was upregulated, leading to the inhibition of gastric cancer cell proliferation and migration (73). By investigating the impact of COR on the migration and invasive ability of liver cancer cells, it was found that COR reduced the expression of C-X-C chemokine receptor type 4 (CXCR4) and significantly inhibited the migration and invasion of liver cancer cells in a dose-dependent manner (74). Furthermore, researchers discovered that COR effectively inhibited the proliferation, wound healing, transwell migration, and tube formation of endothelial cells (75). Endothelial cells play a crucial role in the process of blood vessel formation, implying that COR may inhibit the growth of cancer cells, such as cholangiocarcinoma, by suppressing tumor cell angiogenesis, migration, and proliferation (76). In conclusion, COR can inhibit the invasion and migration of cancer cells through mechanisms involving the suppression of MMPs expression, angiogenesis, inhibition of CXCR4 expression, and induction of CLEC-2 expression.

### Cordycepin's modulation of the immune system in cancer therapy

The immune system plays a critical role in recognizing and eliminating cancer cells, but tumors often develop mechanisms to evade immune surveillance (77). Consequently, there is a growing interest in identifying agents that can enhance the immune response against cancer. The immunomodulatory effects of COR have been extensively investigated, and emerging evidence suggests its ability to regulate various components of the immune system, including immune cells, cytokines, and immune checkpoint molecules (78). Studies have shown that COR not only reduces T cell apoptosis but also facilitates increased infiltration of T cells and leukocyte into tumors (79). Deng et al. (80) have identified a novel mechanism through which COR inhibits the phagocytic immune checkpoint CD47 in tumor cells, thereby promoting the phagocytosis of tumor cells by macrophages. This finding suggests that COR may have immunomodulatory effects and could potentially be utilized as an adjuvant therapy to enhance anti-tumor immune responses.

COR has shown significant potential in modulating the immune system in the context of cancer therapy. By modulating the immune system, COR holds promise in overcoming immune evasion mechanisms employed by cancer cells and enhancing anti-tumor immune responses. However, it is important to note that the specific mechanisms of action of COR still require further research and exploration. Additionally, due to inter-individual variations in tolerance and potential side effects, the rational use of appropriate dosage and treatment duration should be considered. In summary, COR, as a potential anti-tumor substance, holds great research prospects and development value.

## Clinical applications of cordycepin in cancer therapy

COR has shown promising potential in cancer therapy in various clinical applications. Research studies have highlighted its major mediating signaling pathways and therapeutic effects in different types of cancers, including liver cancer (81), lung cancer (82), prostate cancer (83), leukemia (84), brain cancer, and bladder cancer (67) (Table 1). In cancer therapy, COR has been reported to exhibit multiple mechanisms of action. It can inhibit tumor cell proliferation, induce cell cycle arrest, promote apoptosis, and inhibit angiogenesis. Additionally, COR has immunomodulatory effects by enhancing immune responses and regulating immune cells, such as T cells, NK cells, and macrophages.

**Table 1:** Overview of effects and major mediating signaling pathways of cordycepin on various tumor types.

Major mediating signaling pathways	Tumor type	Effects	References
Cysteine-aspartic proteases (Caspase)	Breast cancer	Apoptosis induction	(63)
	Bladder cancer	Apoptosis induction, Anti-proliferation	(67)
	Brain cancer	Apoptosis induction Cell cycle arrest	(68)
	Liver cancer	Apoptosis induction	(81)
	Lung cancer	Apoptosis induction, Anti-proliferation, Anti-metastasis	(82)
	Prostate cancer	Apoptosis induction	(83)
	Leukemia	Apoptosis induction	(84)
Jun NH2-terminal kinases (JNK)	Colon cancer	Cell cycle arrest	(22)
	Oral Cancer	Apoptosis induction	(36)
	Bladder cancer	Cell cycle arrest	(86)
	Lung cancer	Apoptosis induction, Anti-proliferation, Anti-metastasis	(56)
Nuclear factor-κB (NF-κB)	Ovarian cancer	Apoptosis induction	(23)
	Lung cancer	Apoptosis induction, Anti-proliferation	(32)
	Renal cancer	Apoptosis induction	(37)

### Current status of clinical trials involving cordycepin

Surgical resection, chemotherapy, radiation therapy, targeted therapy, and immunotherapy are the main methods used in cancer treatment. However, these individual treatment methods have significant limitations when applied in clinical practice due to their inherent problems, such as strict patient requirements for surgery, the development of drug resistance in cancer cells due to chemotherapy (85), etc. Therefore, combination therapy has become increasingly important in current cancer treatment. T24R2 cells are a DDP-resistant cell line derived from T24 human bladder cancer cells. Research has found that COR induces cell apoptosis through the mitochondrial pathway, thereby increasing the sensitivity of T24R2 cells to DDP. The combination of COR and DDP significantly induces cell death in T24R2 cells (86). Furthermore, COR inhibits osteosarcoma cell growth and invasion and induces osteosarcoma cell apoptosis by activating AMPK and inhibiting the AKT/mTOR signaling pathway and enhances the sensitivity of osteosarcoma cells to DDP (33). Liao (87) found that the combination of COR and *β*-DDP effectively inhibits the proliferation and progression of nasopharyngeal carcinoma in colony formation assays. It has also been found that COR, when combined with paclitaxel or DDP, exhibits a synergistic effect in inhibiting proliferation and promoting apoptosis of non-small cell lung cancer cells, both in the presence and absence of DDP resistance. Specifically, the combination treatment of COR with paclitaxel or DDP not only induces Caspase-mediated apoptosis in cancer cells but also activates the MAPK and P53 signaling pathways (88). These results suggest that the combination of COR with first-line anticancer drugs such as DDP and paclitaxel exhibits superior anti-tumor effects compared to single compounds in terms of inhibiting cell proliferation, inducing cell apoptosis, and regulating the cell cycle. By inducing cell cycle arrest, autophagy, and apoptosis, COR also enhances the radiosensitivity of oral squamous cell carcinoma cells (89).

### Challenges and future prospects for using cordycepin in cancer treatment

While COR has shown potential in cancer treatment, there are still several challenges and future prospects to consider:

#### Standardization and quality control

COR's efficacy can vary depending on the source and extraction method. Ensuring standardized production processes and quality control is essential for consistent therapeutic outcomes.

#### Bioavailability

COR's bioavailability is relatively low, meaning that the body may not absorb or utilize it efficiently. Developing delivery systems or formulations that enhance COR's bioavailability can improve its effectiveness.

#### Drug interactions and safety

COR may interact with other drugs, so careful consideration of potential drug interactions and safety profiles is necessary in clinical applications. Comprehensive safety studies and monitoring are crucial to minimize any adverse effects.

#### Clinical evidence

While preclinical studies and *in vitro* experiments have shown promise, rigorous clinical trials are needed to establish the safety and efficacy of COR in humans. The translation of lab findings to meaningful clinical outcomes remains a critical step.

#### Combination therapy

Considering the complex nature of cancer, combining COR with other treatment modalities, such as chemotherapy, radiation therapy, or immunotherapy, may enhance synergistic effects and overall therapeutic outcomes.

#### Personalized medicine

Cancer is a heterogeneous disease, and individual patients may respond differently to treatments. Identifying biomarkers or genetic signatures that predict patient responsiveness to COR can help in selecting appropriate candidates for personalized treatment strategies.

Despite these challenges, the future prospects for COR in cancer treatment are promising. With further research, clinical trials, and technological advancements, COR may hold potential as an adjunct or alternative therapy option, offering improved outcomes and better quality of life for cancer patients.

## Conclusion

The development of anticancer drugs has always been a focal point in medicine, but results have been limited due to a lack of understanding of cancer mechanisms. Future efforts should intensify basic research on cancer development, cell resistance, and drugs. In recent years, with advancements in *Cordyceps* cultivation, the medicinal value of COR, a nucleoside antibiotic from *Cordyceps militaris*, has gained attention. COR has shown potential as an anticancer agent by inhibiting cell proliferation, inducing apoptosis, suppressing angiogenesis, and inhibiting metastasis, with additional immunomodulatory effects. However, there are still some challenges that need to be addressed before COR can be widely utilized in clinical settings. These include optimizing the dosage and delivery methods, enhancing its bioavailability, and conducting more rigorous clinical trials to validate its efficacy and safety. Despite these challenges, the future prospects of COR in cancer treatment are promising. Its natural origin, targeted action, and multifaceted effects make COR a compelling candidate for further exploration and development. Continued research and clinical trials will pave the way for the integration of COR into standard cancer treatment regimens, providing new options and improved outcomes for cancer patients.
